# Evaluating the Effects of Strategic Use of High Phytase Levels on Growth Performance and Carcass Characteristics of Late-Finishing Pigs Exposed to Limited Floor Space

**DOI:** 10.3390/ani15223280

**Published:** 2025-11-13

**Authors:** Izadora Batista Kuneff, Pete Wilcock, Eric van Heugten

**Affiliations:** 1 Department of Animal Science, North Carolina State University, Raleigh, NC 27607, USA; ibkuneff@ncsu.edu; 2 AB Vista, Marlborough SN8 4AN, UK; petewilcock@feedworksusa.com

**Keywords:** phytase, finishing pigs, hyper-dosing, inositol, floor space, carcass

## Abstract

This study evaluated the effects of high doses of phytase on the growth performance, carcass characteristics, and serum chemistry of late-finishing pigs housed under space-restricted conditions. Pigs (*n* = 375; 94.63 ± 0.61 kg) were randomly assigned to 48 pens, with 7 to 8 pigs per pen, balanced for gilts and barrows. Two phytase doses (control of 2500 FTU/kg or hyper-dose of 5000 FTU/kg) and two space allocation dimensions (adequate with 0.85 m^2^/pig or restricted with 0.66 m^2^/pig) were combined to create a 2 × 2 factorial arrangement (12 replicates per treatment). The three heaviest pigs per pen were marketed on day 28, and the remaining pigs were marketed on day 42. No interactions (*p* > 0.10) were observed between the floor space allowance and phytase supplementation. The body weight, daily gain, and feed intake at the first marketing and for all pigs marketed were reduced (*p* ≤ 0.009) by space restriction, without affecting the gain-to-feed ratio. Space restriction increased serum protein and decreased urea N, and hyper-dosing phytase increased plasma inositol and serum glucose and decreased serum aspartate aminotransferase (*p* < 0.05). The results indicate that space restriction reduced the growth rate, feed intake, and body weight of late-finishing pigs, and that hyper-dosing phytase was not beneficial in improving growth performance regardless of space allowance.

## Simple Summary

The reduction in available floor space per unit of pig body weight reduces growth performance during the late-finishing stage of pig production. Feeding elevated levels of phytase has the potential to destroy the anti-nutrient phytate present in grains and oilseeds, thereby increasing nutrient availability. Applying high doses of phytase in swine diets is a strategy to reduce feed costs and environmental phosphorus pollution, while promoting increased growth performance of pigs. In addition, the associated increase in inositol and other nutrients may improve growth performance and become an important strategy to reduce the stress of heavy weight pigs housed under restricted space. Therefore, this study evaluated the impacts of hyper-dosing dietary phytase (5000 FTU/kg of feed) on the growth performance, carcass characteristics, inositol concentrations, and serum chemistry of late-finishing pigs when housed under adequate or restricted space. Space restriction reduced growth rate, final body weight at marketing, and feed intake, without affecting feed efficiency. Hyper-dosing phytase did not impact growth performance, regardless of space restriction, but it increased inositol concentrations, increased serum glucose concentrations, reduced aspartate aminotransferase, and tended to reduce alanine aminotransferase compared to super-dosing (2500 FTU/kg). Feeding extra phytase did not improve the growth performance of pigs regardless of space restriction.

## 1. Introduction

Limiting the floor space allowance for finishing pigs decreases their growth performance [1,2,3,4,5,6]. Providing the proper space allowance is a balance between maintaining welfare, optimizing growth performance and facility use, and maximizing economic return.

Expressing the space requirements of pigs per unit of body weight is preferred using an allometric approach rather than as space per pig [2]. This suggested approach expresses space requirements as follows: space allowance (m^2^) = 0.0336 × (body weight, kg)^0.667^ [2]. The 0.0336 value is the calculated space allowance coefficient (*k*) determined by the broken-line analysis of average daily gain as a function of the space allowance of grower-finisher pigs on fully slatted floors. Thus, an increase in body weight will lead to greater space requirements. Consequently, as pigs grow in a fixed space, their available area will become restricted, resulting in decreased performance. If the *k* value falls below 0.0336, both weight gain and feed intake will be adversely affected [2]. A meta-analysis [7] estimated that, for each 0.001 unit decline in the *k* value, the daily gain, feed intake, and gain-to-feed ratio in pigs over 125 kg in body weight decreased by 0.88%, 0.58%, and 0.31%, respectively. This issue is especially harmful to the performance of pigs at heavier body weights, such as during the late-finishing period of the growth cycle [1,2,3,4,5,6,7]. The commercial industry has approached this challenge by split marketing, wherein heavy pigs in the pen are marketed first, followed by additional marketing groups during a later time to allow slower growing pigs to reach their target marketing weight. This provides more space for the remaining pigs, which has been demonstrated to improve their growth performance [1,2,4,5,6,7]. Thus, marketing decisions are highly dynamic and need to consider many factors, including feed cost, pig price, and space availability.

The supplementation of phytase in swine diets has traditionally been focused on degrading phytate (a potent anti-nutritional factor), thus increasing phosphorus (P) availability and reducing P excretion in manure. However, in some cases, the supplementation of phytase in P adequate diets has been demonstrated to improve the performance of pigs and poultry, suggesting that improvements in growth performance are not completely explained by an enhanced P availability [8,9,10,11,12]. Several studies in pigs have demonstrated an improved pig performance with high levels of phytase in nursery, grower, and finisher pigs [11,12,13,14,15,16]. Supplementing phytase at super-dosing levels (1500–2500 FTU/kg) promoted the extra-phosphoric effects of phytase in addition to releasing P [8,9,10,11,12,13,14,15,16,17]. These extra-phosphoric effects may be related to the (partial) destruction of phytic acid, thereby improving Ca, Zn, Fe, Mg, and Na utilization, enhancing amino acid and energy utilization, improving the activity of gastric enzymes (pepsin, trypsin, and chymotrypsin), and reducing endogenous losses (enzymes or mucin) [8,9,10,11,12,13,14,15,16,17]. Super-dosing phytase at 1500 to 2500 FTU/kg aims to degrade at least 85% of insoluble anti-nutritional phytate esters and generate myo-inositol, which presumably plays an important role in facilitating the transport of fats and fat-soluble nutrients [18,19,20,21,22,23]. The effect of super-dosing phytase and the generation of myo-inositol have been studied primarily in poultry, later showing benefits in pigs [12,13,19,23,24,25,26]. Furthermore, researchers have also investigated if even higher levels of phytase in swine diets could provide a response in growth performance. There is evidence that phytase continues to improve performance, bone characteristics, and Ca and P digestibility at doses up to 10,000 [24], 12,000 [25], 15,000 [11], and 20,000 FTU/kg [26]. It should be noted that current phytase enzymes dephosphorylate inositol esters more efficiently compared to older versions of phytase [12,19]. For instance, a phytase inclusion above 2500 FTU/kg (hereafter referred to as hyper-dosing) can nearly destroy all the anti-nutrient phytate complex present in grains and oilseed ingredients of pig diets, allowing the release of inositol compounds [19,20,21,22,23,24,25,26,27,28,29,30,31,32,33,34,35,36,37].

Inositol, a carbocyclic-hexane sugar compound of the phytic acid complex, has been considered to play a role in the optimization of growth performance when growing pigs receive higher levels of phytase in their diets [22,25,29,35,37,38,39]. Once absorbed, myo-inositol contributes to intracellular signaling cascades by serving as a precursor for phosphatidylinositol and its phosphorylated derivatives, such as phosphatidylinositol (3,4,5)-trisphosphate (PIP_3_), which are essential components of the insulin-signaling pathway [40]. Indeed, elevated myo-inositol levels have been associated with enhanced insulin sensitivity, improved glucose uptake, and increased anabolic signaling. Furthermore, inositol is associated with increased serotonin and dopamine concentrations, reducing stress levels and increasing feed intake [21,22,30,37]. Thus, we speculate that a supplementation of high levels of phytase could be a practical and economically relevant strategy to improve growth performance during the late-finishing phase when floor space becomes limited [23,28].

Given the decreasing cost of phytase and rising feed ingredient costs, higher levels of supplemental phytase (>2500 FTU/kg) above those typically used with current super-dosing levels may be beneficial if improvements in growth and economic return can be achieved. Work in grower pigs (>30 kg) showed that supplementing incremental doses of phytase up to 8000 FTU/kg resulted in a linear response in portal and peripheral plasma inositol concentrations, suggesting that higher doses of phytase beyond super-dosing may be advantageous in generating inositol in pigs [16]. The late-finishing phase represents a period of increased stress related to decreasing floor space per unit of pig body weight, resulting in a relatively profound reduction in pig performance. Therefore, we hypothesize that specifically targeting the latter portion of the growth phase of finishing pigs (from approximately 100 kg of body weight) with the supplementation of hyper-doses (>2500 FTU/kg) of phytase will improve growth and feed efficiency and will result in higher concentrations of plasma inositol compared to super-dosing. Therefore, this study was designed to evaluate the impacts of the hyper-dosing of phytase (5000 FTU/kg) compared to the relatively common super-dosing levels of phytase (2500 FTU/kg) used in the US swine industry on the growth performance, serum chemistry profile, plasma inositol, and glucose concentrations of late-finishing pigs when housed under either adequate or restricted space allowance.

## 2. Materials and Methods

### 2.1. Experimental Design and Animal Management

**Figure 1:**
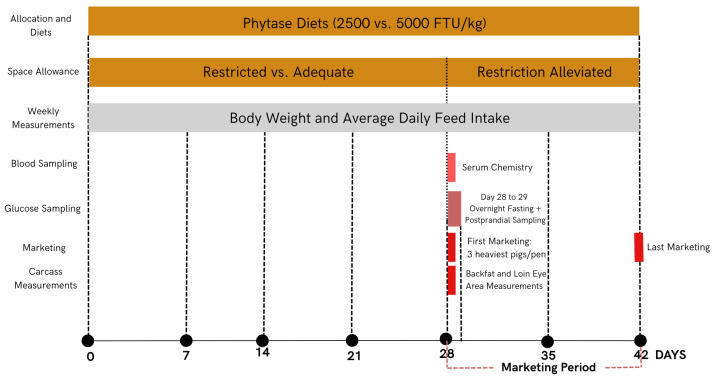
Chronological illustration of experimental design, sampling, and measurements.

A total of 375 late-finishing pigs (Smithfield Premium Genetics; body weight of 94.63 ± 0.61 kg) were randomly assigned to 48 pens, with 7 to 8 pigs per pen. Pigs were equally balanced for gilts and barrows within pens. Two space allocation dimensions (adequate with 0.85 m^2^/pig or restricted with 0.66 m^2^/pig) and two phytase supplementation doses (control of 2500 FTU/kg or hyper-dose of 5000 FTU/kg of an *Escherichia coli* 6-phytase expressed in *Trichoderma reesei* and commercialized as Quantum Blue 10K Phytase by AB Vista, Marlborough, UK) were combined to create four treatments in a 2 × 2 factorial arrangement (twelve replicates per treatment). The space allowances used were determined based on an allometric equation [2] represented as follows: space allowance (m^2^) = 0.0336 × (body weight (kg))^0.667^. Based on this approach, the adequate space allowance was calculated based on the target marketing weight of 130 kg, such that space was always adequate until pigs reached this market weight. The pen dimensions of 2.95 by 2.39 m and feeder dimensions of 38 by 73 cm were considered in the calculation of the space allowance. For the restricted space, space allowance was calculated based on the starting weight of the pigs (90 kg) such that pig space was always restricted for the entire duration of the study. To accomplish the targeted restricted space of 0.66 m^2^, the back gate of the pens was moved inward to reduce the total pen area. Each pen used in this study had four fully accessible drinkers and a self-feeder that provided two feeding spaces. If pig mortality occurred, the pen dimension was adjusted to maintain the targeted space allowance per pig, and this was performed only for the restricted space treatment. Space restriction was employed for a total of 28 days. On day 28, three of the heaviest pigs were removed from each pen for marketing purposes to mimic commercial marketing practices. The remaining pigs continued in the test for an additional 2 weeks to determine the effects of alleviating the space restriction and were then marketed on day 42 of the study (Figure 1).

### 2.2. Experimental Diets and Manufacturing

Pigs were fed a finishing diet primarily based on corn and soybean meal as major ingredients (Table 1). Diets were formulated to contain 0.68% standardized ileal digestible (SID) lysine, 0.55% Ca, and 0.24% available P and met or exceeded nutrient requirement estimates [41]. These formulated values included phytase nutrient matrix values for Ca (0.16%), P (0.15%), SID amino acids, and net energy according to manufacturer recommendations, and it was the same for both supplementation levels. Experimental diets were manufactured at the North Carolina State University Feed Mill Education Unit located in Raleigh, NC. Corn was ground with a hammer mill to obtain a grind size of approximately 600 to 800 microns. Dry ingredients were blended in a double ribbon mixer and poultry fat was added after the dry mixing was complete to mix the final diets. Food coloring in a matrix of corn grits (Microgrits, Micro-Tracers, San Francisco, CA, USA) was used to distinguish diets for visual verification. Diets were transported and placed into individual bins located at the research facility for feeding. At the time of feeding, feed was deposited into 60 kg capacity bins, weighed, and then added to the self-feeders. Feed samples were collected from the bins at the time of feeding throughout the study and combined within treatment to obtain representative samples. Samples were analyzed in duplicate for proximate components (moisture, crude protein, crude fat, crude fiber, and ash) and Ca and P by the University of Missouri Agricultural Experiment Station Chemical Laboratories (Columbia, MO, USA). Additionally, samples were analyzed in duplicate for phytase activity (AB Vista, Marlborough, UK).

### 2.3. Sampling and Measurements

Pigs were monitored twice daily for signs of illness, injury, or undue stress. Pigs were individually weighed on days 0, 7, 14, 21, 28, 35, and 42. After data collection on day 28, the three heaviest pigs (two barrows and one gilt) of each pen were removed from the pens for marketing at a local processing plant. This constituted the first marketing event and the end of the space restriction. The remaining pigs (four to five pigs per pen) were marketed for the final marketing event on day 42. This process allowed for the determination of the impact of space restriction (up to day 28) and subsequent alleviation of space restriction by removing the heaviest pigs from day 28 to 42. Average daily gain (ADG) was calculated for each period as the difference in body weight between periods, divided by the number of days in the period. In addition, the average body weight and ADG of all pigs marketed were calculated and included pigs marketed on day 28 and day 42. At the time of weighing, feed remaining in the feeders was measured, and feed disappearance (ADFI) was calculated from the total feed added to the feeders minus the feed remaining in the feeders divided by the number of pigs and the number of days in the period. Gain-to-feed ratio was calculated by dividing total gain by total feed for each period and for all pigs marketed. Incidences of mortality and medical treatments were recorded. In cases where mortality occurred, growth performance measurements for the affected pen were calculated based on the total number of days pigs were present in the pen for each period.

Backfat and loin eye area were measured on all pigs on day 28 using real-time ultrasound (Aloka, Tokyo, Japan, SSD 500 V, probe 3.5 MHz; size 5011), using software by Biotronics (BioSoft Toolbox II for Swine 3.0.0.0 2007–2018). Ultrasound (US) measurements were taken between the 10th and 11th ribs at approximately 4 cm distance from the back central line.

Blood samples of two barrows (the lightest and the heaviest body weight barrow within each pen) were obtained for the collection of serum on day 28 by jugular venipuncture using vacuum tubes (BD Vacutainer; Becton, Dickinson and Company, Franklin Lakes, NJ, USA). Additional blood samples were obtained for the collection of plasma from the same pigs on day 28 using vacuum tubes (BD Vacutainer, spray-coated lithium heparin as anti-coagulant). Blood samples were centrifuged at 1000× *g* for 15 min for the collection of serum and plasma.

Serum samples were submitted to Antech Diagnostics Laboratory (Fountain Valley, CA, USA) for the analysis of total protein, albumin, globulin, aspartate aminotransferase, alanine aminotransferase, alkaline phosphatase, urea nitrogen, creatinine, glucose, phosphorus, calcium, magnesium, sodium, potassium, chloride, cholesterol, creatine kinase, ϒ-glutamyl transferase, triglycerides, amylase, and pancreas specific lipase. The analysis used the Immulite automated system (Siemens Healthcare Diagnostics, Deerfield, IL, USA) consisting of an enzyme-amplified chemiluminescent technology.

Plasma samples were analyzed for inositol concentrations. A volume of 0.5 mL of plasma was transferred into an empty tube (Eppendorf, Hamburg, Germany) containing 0.5 mL of 1 N of perchloric acid. Samples were vortexed and kept at 4 °C for 30 min. Thereafter, samples were centrifuged at 4 °C in an ultra-high-speed centrifuge at 17,500× *g* for 10 min. After centrifugation, approximately 1 mL of the supernatant was collected with a disposable syringe attached to a syringe filter (PTFE, 0.45 µm, 13 mm diameter; Fisher Scientific, Cork, Ireland). Samples were then submitted to AB Vista (Marlborough, UK) for inositol analysis by reversed-phase HPLC using a Dionex, Sunnyvale, CA, USA, DX600 system with subsequent mass spectrometry according to methods described previously [16,23].

At the time of the first marketing (on day 28 after completion of all measurements), the heaviest barrow from each pen (*n* = 48 pigs) that was identified for marketing was moved to an individual pen and fasted overnight for 16 h for postprandial glucose analysis. Post-fast, each pig was fed its originally assigned dietary treatment. The amount of feed provided to pigs after the overnight fast was restricted to 106 kcal ME per kg BW^0.75^, using individual BW of each pig [41]. Half of this amount was provided to each pig as the morning meal prior to collecting blood samples. Exactly 45 min after feeding, blood samples were collected via venipuncture using vacuum tubes (spray-coated lithium heparin as anticoagulant) to obtain plasma for glucose analysis using UV-spectrometry [42]. Any remaining feed not consumed after 45 min was measured after blood samples were collected. Blood samples were centrifuged to obtain plasma, aliquoted in duplicate, and transferred to cryogenic vials and stored at −80 °C until further analysis. Plasma concentrations of glucose were quantified enzymatically using a glucose analysis kit (Sigma #GAHK-20, Sigma-Aldrich, Inc., St. Louis, MO, USA) following the manufacturer’s instructions [42].

### 2.4. Statistical Analyses

All data were analyzed using general linear model procedures (PROC GLM) of SAS 9.4 (SAS Institute, Cary, NC, USA) as a completely randomized design with factorial arrangement of treatments. The model included the main effects of space restriction (adequate or restricted), phytase supplementation (2500 or 5000 FTU/kg), and their interaction. Pens were considered the experimental unit. Initial body weight was used as a covariate for the analysis of growth performance. Body weight on day 28 was used as a covariate for the analysis of carcass characteristics. Differences between treatments were evaluated using the Least Significant Difference procedure. Statistically significant differences were considered at *p* ≤ 0.05, with tendencies at 0.05 < *p* ≤ 0.10. The initial inositol concentration on day 0 was used as a covariate in the statistical analysis of inositol concentrations measured on day 28. For postprandial plasma glucose analysis, pigs did not consume all of their feed prior to the collection of blood samples; therefore, the amount of feed consumed prior to collecting blood samples was used as a covariate in the analysis of plasma glucose concentrations.

## 3. Results

### 3.1. Analysis of Experimental Diets

The analyzed concentrations of phytase were 2970 and 6560 FTU/kg of complete feed for the 2500 and 5000 FTU/kg diets (Table 1). Although these values were slightly above the planned inclusion levels, they are within a reasonable expected range to allow comparisons between dietary treatments. Similarly, proximate analyses and Ca and P analytical results were generally in good agreement with the calculated concentrations.

### 3.2. Growth Performance, Feed Efficiency, and Carcass Characteristics

The average mortality in the present study was 1.87%, representing four pigs in the restricted and three pigs in the adequate space groups, respectively. The total number of medical treatment days (number of pigs multiplied by the number of days treated) were 13 and 28 for pigs under restricted versus adequate space, respectively. No interactions were observed between the floor space allowance and phytase supplementation (*p* > 0.10). The restriction of space reduced pig BW (*p* ≤ 0.007) on days 21 and 28 of the study (Table 2) and tended to reduce BW on day 35 and 42 (*p* ≤ 0.078). Overall, restricted space resulted in a reduction in body weight for all pigs marketed (*p* = 0.009) by 2.07 kg. Space restriction reduced ADG for the first (*p* = 0.045) and third week (*p* = 0.001), and it reduced ADG for the first marketing group and for all pigs marketed (*p* ≤ 0.001). The average daily feed intake (ADFI) was reduced for each week (*p* ≤ 0.002) for space restricted pigs until the first marketing event on day (28 days).

For the 2 weeks after the first marketing, ADFI was reduced (2.9%; *p* = 0.038) due to the prior space restriction, but to a lesser extent compared to the first marketing (6.3% reduction in feed intake). Considering all pigs marketed, ADFI was reduced in space restricted pigs by 6.2% (*p* < 0.001). Space restriction tended to reduce the gain-to-feed ratio on the third week of the study (*p* = 0.078) but had no effect during any other time period (*p* ≥ 0.168). The carcass characteristics measured on day 28 (for all pigs) using ultrasound were analyzed using body weight on day 28 as a covariate. Backfat depth tended to be reduced by 4% (*p* = 0.062) when pigs were housed in restricted space compared to adequate space, but loin eye area was not affected (Table 2). The supplementation of 5000 FTU/kg of phytase did not impact the overall body weight for all pigs marketed (*p* = 0.194; Table 2), but there was a tendency (*p* < 0.10) for a lower BW on day 14, 35, and 42. The average daily gain, ADFI, and gain-to-feed ratio for the weekly periods, the first marketing group, the second marketing group, or the overall pigs marketed were not affected by additional phytase supplementation (*p* > 0.220). The supplementation of 5000 FTU/kg of phytase did not affect carcass characteristics.

### 3.3. Serum Chemistry

There were no significant interactions (*p* > 0.10) between space and diet. Space restriction tended to increase (*p* < 0.10) serum concentrations of total protein, aspartate aminotransferase, and triglycerides (Table 3). Hyper-dosing phytase decreased serum aspartate aminotransferase (*p* = 0.038), increased serum glucose concentrations (*p* = 0.051), and tended to reduce alanine aminotransferase concentrations (*p* = 0.070).

### 3.4. Inositol Analysis

The initial plasma inositol concentration determined on day 0 was used as a covariate in the statistical analysis of inositol concentrations on day 28 of the study. The average plasma inositol concentration on day 0 was 44.24 µM. Space restriction did not affect (*p* > 0.10) inositol concentrations (Table 4). Feeding the hyper-dosed level of phytase increased inositol concentrations by 22.6% compared to super-dosed diets (*p* = 0.003).

### 3.5. Glucose Analysis

Glucose concentrations were determined in pigs exactly 45 min after being fed a single limited meal following an overnight fast. The pigs did not consume all of their feed prior to the collection of blood samples; therefore, the amount of feed consumed prior to collection was used as a covariate in the analysis of serum glucose concentrations. Space restriction and the supplementation of phytase above super-dosing levels did not impact (*p* > 0.799) postprandial serum glucose concentrations (Table 4).

## 4. Discussion

The primary objective of this study was to investigate the effects of the hyper-dosing (5000 FTU/kg) of phytase compared to super-dosing (2500 FTU/kg) on the growth performance of late-finishing pigs housed under restricted or adequate floor space. We specifically used a 2500 FTU/kg phytase supplementation dose as the control because it has already been demonstrated to be effective in improving growth and feed efficiency, and it is considered a standard practice in many commercial operations [43,44]. We hypothesized that space restriction would negatively impact growth, while a supplementation with higher levels of phytase would mitigate some of this impact by increasing plasma inositol concentrations, which in turn might improve growth performance. Additionally, we specifically aimed to evaluate the interaction between space allowance and dietary interventions particularly during the late-finishing phase, which is a period characterized by rapid growth and increased susceptibility to stress from limited space.

Pigs under restricted space conditions had markedly reduced growth rates, which aligns with the general consensus that over-crowding leads to an increased competition for resources such as feed and space, as well as higher stress levels, all of which can inhibit growth [1,2,3,4,5,6,7]. Space restriction in the present study resulted in a reduction in body weight of 1.88 kg after the 28-day space restricted period. When space restriction was alleviated by marketing the three heaviest pigs in each pen, the remaining pigs gained weight at a similar rate compared to the non-restricted pigs; however, the negative effect of the original space restriction remained evident. When considering the average market weight of all pigs (those marketed at 28 days and the ones marketed after 42 days), space-restricted pigs were 2.07 kg lighter than pigs that were not restricted. The decline in growth under space restriction was consistent with the predicted effects based on allometric equations for space allowance, where the space requirements increase as pig body weight rises [2,3]. In the present study, the space allowance coefficient (*k* value) was based on the final body weight for the adequately housed pigs and the initial body weight for the restricted pigs. The calculated *k* values based on the actual body weight of pigs at the first marketing event were 0.0339 and 0.0266 for adequate and restricted space, respectively. Thus, pigs housed under adequate conditions were never below the *k* value, whereas pigs housed under the restricted conditions were always below the *k* value. As a result, the available space per pig in the restricted group became more limited as their body weight increased. This is consistent with the progressive reductions in body weight observed over time between restricted and adequate pigs in the present study. This also aligns with earlier research, which highlighted that reduced space, particularly during the later stages of growth, severely compromised pig performance [1,2]. Feed intake was reduced throughout the study, showing an overall reduction in the feed intake of space-restricted pigs of 6.25% (for all pigs marketed). This compares to a reduction in the daily gain of pigs housed in restricted space of 5.4% and no apparent differences in the gain-to-feed ratio, suggesting that the reduced feed intake was the main factor in reducing growth.

Super-dosing phytase (typically 2500 FTU/kg) has been widely reported to enhance growth performance, feed efficiency, and nutrient digestibility by promoting the breakdown of the anti-nutrient phytate, which limits the negative impacts of phytate [45] and releases bioavailable myo-inositol. Inositol, in turn, is believed to contribute to cell signaling, muscle development, fat metabolism, and insulin-like effects in mammals, and is associated with increases in serotonin and dopamine and a reduction in stress levels, all of which could potentially improve growth performance [9,10,30,31]. While phytase supplementation at levels greater than those required for P release is well-documented and increasingly common in the swine industry, particularly in the U.S. [12,19,23], there is still debate about whether higher dosages (e.g., 5000 FTU/kg) provide additional benefits. The rationale for investigating doses above 2500 FTU/kg is based on the premise that modern phytase formulations continue to be efficient at nearly completely degrading phytate at these higher doses and generating myo-inositol, which may offer further improvements in growth performance beyond what is achieved with super-dosing levels. Positive linear responses in portal and peripheral blood plasma inositol have been reported in grower pigs supplemented with increasing incremental doses of phytase up to 8000 FTU/kg [16]. In our previous work under commercial field conditions, the dosing of phytase at 3000, 4500, or 6000 FTU/kg linearly improved daily gain and feed efficiency, and this improvement was most dramatic during the final finishing phase (95 to 125 kg of body weight), improving feed efficiency from 3.76 to 3.41 (9.3% improvement) [46]. This final finishing phase represents a period of increased stress related to decreasing floor space per unit of pig body weight, resulting in a relatively profound reduction in pig performance. When applying a similar restricted floor space allowance to late-finishing pigs in the present study, providing 5000 FTU/kg of phytase did not improve pig performance when compared to the standard 2500 FTU/kg supplementation level.

The lack of growth improvement with 5000 FTU/kg of phytase supplementation under both restricted and adequate space conditions in the present study suggests that 2500 FTU/kg may already be near the threshold for optimal phytate degradation, with little additional benefit gained from increasing the dose. At 2500 FTU/kg, phytase supplementation has been shown to achieve up to an 85% degradation of phytate, and increasing the dose further may not significantly enhance this effect [17,18,19,20,21,22,23,45]. However, this is in contrast with our observation showing that hyper-dosing phytase increased serum inositol concentrations by 22.6% when measured at the peak of space restriction, suggesting that additional phytase was still effective in breaking down phytic acid and its derivatives. Previous work reported that the dose-dependent relationship between phytase supplementation and growth performance diminished beyond 2500 FTU/kg [13,19,23,36]. Similarly, in the present study, no improvements in pig performance were evident, and therefore the hyper-dosing of phytase was not effective. In contrast, we previously reported linear improvements in average daily gain and feed efficiency with supplemental phytase up to 6000 FTU/kg, and this improvement was most dramatic during the final finishing phase (95 to 125 kg of body weight). The lack of growth response in the present study suggests that myo-inositol alone may not be the primary driver of growth enhancement in late-finisher pigs, unlike nursery pigs where researchers showed that inositol could be considered as a conditionally essential nutrient [37]. Other factors, including the extent of phytate hydrolysis, dietary protein availability, intestinal absorption efficiency, or diminishing returns of inositol effects, may play a role in modulating growth performance in response to phytase supplementation. In poultry, myo-inositol has been linked to the growth-promoting effects of phytase, whereas its role in pigs appears to be more complex and context-dependent [47,48,49,50]. Nonetheless, research in newly weaned pigs [37] demonstrated an improved growth performance with super-dosing phytase, which was directly related to myo-inositol production and showed that myo-inositol had a greater metabolic impact in piglets immediately after weaning, which is a very challenging and stressful period. This suggests that the impact of phytase supplementation may be age-dependent and likely varies depending on the stress model used.

A serum chemistry analysis was conducted to provide a broad evaluation of hepatic, renal, and metabolic responses to space restriction and high phytase inclusion and to capture potential metabolic effects related to phytate degradation and myo-inositol release. The results showed reduced serum aspartate aminotransferase concentrations, slightly decreased alanine aminotransferase concentrations, and increased serum glucose concentrations due to phytase supplementation at 5000 FTU/kg. These changes agree with previous reports suggesting that phytase supplementation may modulate hepatic function and energy metabolism through the release of myo-inositol from phytate degradation in newly weaned pigs and broiler chickens [47,49,50,51], as well as potentially improving antioxidant status [52], supporting liver function. High levels of AST and ALT are indicators of hepatotoxicity and are generally considered to be biologically relevant to liver damage when their values are at least 3 times beyond the upper normal range [53]. In general, all serum chemistry values in the present study were within reported reference ranges [54], indicating that there were no biologically relevant effects of space restriction or phytase supplementation. The postprandial glucose concentrations measured 45 min after feeding did not differ across space restriction or phytase supplementation levels, indicating that these factors did not acutely influence glucose homeostasis, similar to the observations in poultry [50]. This aligns with previous research suggesting that, while phytase-derived myo-inositol influences glucose metabolism through increased insulin sensitivity, its impact on immediate postprandial glucose levels appears to be inconsistent in pigs [33].

## 5. Conclusions

It can be concluded that restricting space in late-finishing pigs negatively impacted growth, and this was attributed to a reduction in feed intake with no effects on feed efficiency. Hyper-dosing phytase (5000 FTU/kg) increased circulating myo-inositol concentrations but did not further improve growth performance or feed efficiency relative to 2500 FTU/kg. These findings indicate that late-finishing pigs likely reach a plateau in phytase responsiveness at or below 2500 FTU/kg, with higher inclusion rates offering no additional performance benefits under either adequate or restricted space conditions. Future research should investigate whether higher phytase levels may play a role in a more health-challenged environment in this late-finisher period, potentially evaluating incrementally higher doses of phytase and myo-inositol on the functional impacts they may provide beyond growth, including stress and immune responses.

## Figures and Tables

**Table 1:** Composition of the experimental diets (as-fed basis, %).

Ingredient	Phytase	
	2500 FTU/kg	5000 FTU/kg
Corn, yellow dent	86.70	86.67
Soybean meal, 47.5% CP	10.20	10.20
Poultry fat	1.000	1.000
L-lysine·HCl	0.290	0.290
DL-methionine	0.001	0.001
L-threonine	0.064	0.064
L-tryptophan	0.018	0.018
Monocalcium phosphate, 21% P	0.191	0.191
Limestone	0.852	0.852
Salt	0.400	0.400
Finisher trace mineral premix ^1^	0.150	0.150
Finisher vitamin premix ^2^	0.050	0.050
Phytase ^3^	0.025	0.050
Xylanase ^4^	0.010	0.010
Colorant ^5^	0.050	0.050
***Calculated Composition***		
Metabolizable energy, kcal/kg	3406	3406
Net energy, kcal/kg	2604	2604
Crude protein, %	12.23	12.23
Total lysine, %	0.770	0.770
Calcium, % ^6^	0.551	0.551
Total phosphorus, %	0.335	0.335
Available phosphorus, % ^6^	0.240	0.240
***Standardized ileal digestible amino acids* ^6^**		
Lys, %	0.680	0.680
Thr, %	0.449	0.449
Met, %	0.198	0.198
Met + Cys, %	0.408	0.408
Trp, %	0.129	0.129
Ile, %	0.425	0.425
Val, %	0.502	0.502
***Analyzed Composition***		
Phytase, FTU/kg	2970	6560
Crude protein, %	11.86	12.51
Calcium, %	0.610	0.480
Total phosphorus, %	0.340	0.320

^1^ The finisher trace mineral premix provided the following nutrients per kilogram of complete feed: Mn (from manganous oxide), 33 ppm; Zn (from zinc sulfate), 110 ppm; Fe (from ferrous sulfate), 110 ppm; Cu (from copper sulfate), 16.5 ppm; I (from calcium iodate), 0.30 ppm; Se (from sodium selenite), 0.30 ppm. ^2^ The finisher swine vitamin premix provided the following nutrients per kilogram of complete feed: vitamin A, 8267 IU; vitamin D_3_, 1653 IU; vitamin E, 33.0 IU; vitamin B_12_, 44.1 µg; menadione, 3.3 mg; riboflavin, 7.7 mg; d-pantothenic acid, 25.0 mg; niacin, 44.1 mg; biotin, 0.11 mg. ^3^ *Escherichia coli* 6-phytase expressed in *Trichoderma reesei* (Quantum Blue 10K, AB Vista, Marlborough, UK). ^4^ AB Vista, Marlborough, UK. ^5^ To color-code diets (Microgrits, Micro-Tracers, San Francisco, CA, USA). ^6^ Values include phytase nutrient matrix values for Ca (0.16%), P (0.15%), SID amino acids, and net energy.

**Table 2:** Growth performance and carcass characteristics of late-finishing pigs housed in adequate or restricted space and fed different levels of phytase ^1^.

Phytase, FTU/kg	Adequate (0.85 m^2^/Pig)		Restricted (0.66 m^2^/Pig)		SEM	*p* Value	
	2500	5000	2500	5000		Diet	Space
Body Weight, kg							
Day 0	94.57	94.27	94.02	94.82	*	*	*
Day 7	102.70	102.14	102.11	101.87	0.224	0.195	0.164
Day 14	110.84	109.99	110.25	109.38	0.336	0.064	0.194
Day 21	118.61	118.09	117.24	116.23	0.367	0.132	0.002
Day 28	125.55	125.32	124.04	123.07	0.480	0.361	0.007
Day 35	129.33	126.34	126.18	124.86	0.918	0.091	0.071
Day 42	136.63	134.00	133.90	132.06	0.953	0.089	0.078
Overall Marketed	135.62	135.10	134.03	132.56	0.559	0.194	0.009
Average daily gain, kg							
Day 0 to 7	1.162	1.083	1.047	1.042	0.028	0.282	0.045
Day 7 to 14	1.144	1.121	1.107	1.083	0.034	0.609	0.427
Day 14 to 21	1.110	1.161	1.000	1.005	0.027	0.441	0.001
Day 21 to 28	0.998	1.038	0.978	0.986	0.044	0.685	0.549
Day 28 to 35	1.306	1.235	1.270	1.177	0.061	0.321	0.571
Day 35 to 42	1.069	1.084	1.094	1.029	0.035	0.578	0.746
Day 0 to 28	1.104	1.102	1.034	1.032	0.014	0.919	0.001
Day 28 to 42	1.190	1.159	1.182	1.099	0.034	0.220	0.462
Day 0 to 42	1.125	1.121	1.070	1.054	0.013	0.568	0.001
Average daily feed intake, kg							
Day 0 to 7	3.340	3.364	3.190	3.220	0.034	0.578	0.002
Day 7 to 14	3.531	3.495	3.329	3.330	0.038	0.713	0.001
Day 14 to 21	3.530	3.571	3.315	3.330	0.036	0.564	<0.001
Day 21 to 28	3.670	3.630	3.210	3.383	0.050	0.342	<0.001
Day 28 to 35	3.797	3.736	3.609	3.511	0.078	0.456	0.058
Day 35 to 42	3.745	3.742	3.606	3.510	0.060	0.547	0.027
Day 0 to 28	3.510	3.496	3.257	3.311	0.030	0.664	<0.001
Day 28 to 42	3.768	3.690	3.610	3.507	0.059	0.258	0.038
Day 0 to 42	3.574	3.563	3.337	3.359	0.034	0.908	<0.001
Gain-to-feed ratio, kg/kg							
Day 0 to 7	0.349	0.332	0.327	0.325	0.007	0.371	0.168
Day 7 to 14	0.325	0.322	0.335	0.325	0.009	0.586	0.631
Day 14 to 21	0.315	0.326	0.302	0.302	0.008	0.601	0.078
Day 21 to 28	0.273	0.286	0.304	0.295	0.011	0.907	0.205
Day 28 to 35	0.344	0.328	0.351	0.339	0.014	0.466	0.660
Day 35 to 42	0.284	0.286	0.303	0.292	0.008	0.663	0.231
Day 0 to 28	0.315	0.315	0.321	0.314	0.003	0.408	0.622
Day 28 to 42	0.316	0.309	0.326	0.317	0.009	0.339	0.302
Day 0 to 42	0.315	0.315	0.321	0.314	0.004	0.408	0.622
Carcass characteristics							
Back fat thickness, cm	2.876	2.791	2.689	2.760	0.043	0.878	0.062
Loin eye area, mm^2^	66.28	66.65	65.93	67.60	0.497	0.140	0.655

^1^ Values represent least square means of 48 pens with seven to eight pigs per pen. * Growth performance data were analyzed by using initial body weight (day 0) as a covariate. The period of day 0 to 28 represents the period of space restriction before marketing the heaviest three pigs in each pen. The period of day 28 to 42 represents the subsequent alleviation of space restriction after removing the three heaviest pigs. The overall period of day 0 to 42 considered all pigs that were marketed (day 28 and 42). Carcass characteristics were measured by ultrasound in all pigs on day 28 and final body weight was used as a covariate. There were no statistically significant interactive effects between space and diet for any of the dependent variables (*p* ≥ 0.108).

**Table 3:** Serum chemistry panel of late-finishing pigs housed in adequate or restricted space and fed different levels of phytase ^1^.

Phytase, FTU/kg	Adequate (0.85 m^2^/Pig)		Restricted (0.66 m^2^/Pig)		SEM	*p* Value	
	2500	5000	2500	5000		Diet	Space
Total protein, g/dL	6.65	6.58	6.87	6.68	0.09	0.156	0.076
Albumin, g/dL	3.61	3.49	3.60	3.54	0.09	0.309	0.868
Globulin, g/dL	3.03	3.09	3.28	3.14	0.13	0.736	0.227
Aspartate aminotransferase, IU/L	36.16	32.65	59.95	33.91	7.19	0.038	0.078
Alanine aminotransferase, IU/L	39.27	36.36	40.36	38.65	1.29	0.070	0.185
Alkaline phosphatase, IU/L	151.65	149.85	153.06	138.85	8.67	0.347	0.573
Serum urea nitrogen, mg/dL	9.71	10.05	9.34	9.17	0.42	0.841	0.134
Creatinine, mg/dL	1.55	1.55	1.55	1.53	0.04	0.681	0.758
Glucose, mg/dL	83.68	87.27	83.31	86.43	1.74	0.051	0.723
P, mg/dL	7.78 ^x^	8.10 ^y^	8.01 ^xy^	7.81 ^x^	0.12	0.617	0.748
Ca, mg/dL	10.50	10.48	10.44	10.38	0.09	0.636	0.380
Mg, mEq/L	1.56	1.50	1.55	1.55	0.02	0.213	0.315
Na, mEq/L	144.43	144.47	143.80	144.47	0.38	0.347	0.406
K, mEq/L	5.52	5.71	5.75	5.80	0.14	0.373	0.244
Cl, mEq/L	100.30	100.22	100.18	100.59	0.32	0.597	0.692
Cholesterol, mg/dL	98.37	96.87	97.99	93.66	2.73	0.276	0.502
Creatinine phosphokinase, IU/L	1530	1819	3505	1705	588	0.192	0.108
ϒ-glutamyl transferase, IU/L	32.97	33.18	30.73	33.01	3.21	0.693	0.702
Triglyceride, mg/dL	36.15	36.62	41.70	41.33	3.09	0.987	0.093
Amylase, IU/L	1162	1084	1164	1087	59.03	0.184	0.960
Pancreas specific lipase, U/L	8.58	7.39	8.51	7.76	0.61	0.109	0.802

^1^ Values represent least square means of individual pigs (*n* = 96) representing the lightest and the heaviest body weight barrows within each of the 48 pens. Serum chemistry analysis was determined on day 28 of space restriction, immediately before the first marketing event. Interactions between space restriction and phytase supplementation were not significant (*p* > 0.072), except for phosphorus (*p* = 0.026). ^xy^ Means for phosphorus without a common superscript tended to be different (*p* < 0.076).

**Table 4:** Serum concentrations of inositol and glucose in late-finishing pigs provided with adequate (0.85 m^2^/pig) or restricted (0.66 m^2^/pig) floor space and supplemented with dietary phytase.

Phytase, FTU/kg	Adequate (0.85 m^2^/Pig)		Restricted (0.66 m^2^/Pig)		SEM	*p* Value	
	2500	5000	2500	5000		Diet	Space
Inositol, µM ^1^	40.61	55.42	43.97	48.60	3.18	0.003	0.594
Glucose, mg/dL ^2^	111.27	104.76	104.88	113.91	3.80	0.813	0.799

^1^ Serum inositol concentrations were determined in one heavy- and one light-weight pig per pen (48 pens) on day 28 of space restriction. ^2^ Serum concentrations of glucose were determined in 48 pigs (1 pig per pen) exactly 45 min after being fed a restricted meal following an overnight fast. Interactions between space restriction and phytase supplementation were not significant (*p* > 0.114).

## Data Availability

Data associated with the current study are available from the corresponding author upon reasonable request.
